# Dynamic Interplay of Spectrosome and Centrosome Organelles in Asymmetric Stem Cell Divisions

**DOI:** 10.1371/journal.pone.0123294

**Published:** 2015-04-07

**Authors:** Chi Bang, Jun Cheng

**Affiliations:** Department of Bioengineering, The University of Illinois at Chicago, Chicago, Illinois, United States of America; Duke-NUS Graduate Medical School Singapore, SINGAPORE

## Abstract

Stem cells have remarkable self-renewal ability and differentiation potency, which are critical for tissue repair and tissue homeostasis. Recently it has been found, in many systems (e.g. gut, neurons, and hematopoietic stem cells), that the self-renewal and differentiation balance is maintained when the stem cells divide asymmetrically. *Drosophila* male germline stem cells (GSCs), one of the best characterized model systems with well-defined stem cell niches, were reported to divide asymmetrically, where centrosome plays an important role. Utilizing time-lapse live cell imaging, customized tracking, and image processing programs, we found that most acentrosomal GSCs have the spectrosomes reposition from the basal end (wild type) to the apical end close to hub-GSC interface (acentrosomal GSCs). In addition, these apically positioned spectrosomes were mostly stationary while the basally positioned spectrosomes were mobile. For acentrosomal GSCs, their mitotic spindles were still highly oriented and divided asymmetrically with longer mitosis duration, resulting in asymmetric divisions. Moreover, when the spectrosome was knocked out, the centrosomes velocity decreased and centrosomes located closer to hub-GSC interface. We propose that in male GSCs, the spectrosome recruited to the apical end plays a complimentary role in ensuring proper spindle orientation when centrosome function is compromised.

## Introduction

Many stem cells achieve tissue homeostasis through asymmetric stem cell division, effectively balancing the self-renewal ability and differentiation potential [1]. In these systems, imbalance of the stem cell fates could either lead to uncontrolled tumorienesis due to excessive self-renewal [2] or tissue degeneration/aging due to excessive differentiation or reduced self-renewal ability [3, 4]. Some of these stem cells achieve this balance by residing inside a specialized micro-environment (thereafter referred as stem cell niche) that provides cues and signals necessary to the stem cells for maintaining their stem cell identity [5]. Cells leaving the niche, deprived of the cues and signals, would lose the stem cell identity and begin differentiating.

*Drosophila* male germline stem cells (GSCs) are among the best models to study stem cells inside the niche because of well-characterized signaling mechanisms as well as easily-recognized niche structure. Localized at the tip of the testis, the hub cells, residing at the center of the niche, are surrounded by GSCs and cyst stem cells (CySCs). Hub cells secrete unpaired (Upd) factor, which initiates Janus kinase–signal transducers and activators of transcription (JAK-STAT) pathway in GSCs [6–8]. GSCs undergo asymmetric stem cell divisions through stereotypically oriented mitotic spindle relative to hub cells, resulting in one attached and another detached daughter cell to the hub cells [9]. The daughter cell attached to hub cells remains as stem cell, while the other daughter cell displaced from the hub commits to differentiation. Furthermore, the stereotypical mitotic spindle orientation is ensured by the centrosome orientation [9, 10]. Additionally, it was recently found that centrosome orientation plays an important role in the cell cycle progression. A mechanism, known as the centrosome orientation checkpoint, monitors the proper centrosome orientation [11]. If centrosome(s) is not properly oriented, the GSC mitosis is delayed until a correction is made. Centrosomin (cnn) and *Drosophila* E-cadherin are reported to be involved in this centrosome orientation checkpoint [12].

Spectrosome, a spherical cytoskeletal organelle, was initially described in female GSCs in *Drosophila melanogaster* [13], and similar spectrosomal structure was found in certain mammalian lymphocytes [14]. Spectrosome is reported as endoplasmic reticulum-derived, containing proteins such as adducin, ankyrin, cadherin, and spectrin that have binding affinity to microtubules and actin [15–19]. In female GSCs, several reports show that the spectrosome plays an important role in orienting mitotic spindles through interaction with spindle poles [13, 20]. Thus when the spectrosome was knocked out by huli-tai shao (*hts* ^*1*^ *)* mutation, the spindle orientation was severely compromised and randomly oriented [20]. In addition, fusome, a derivative of the spectrosome, was also shown to orient the spindle of later stage spermatocytes [13, 21].

In this study, we show that both centrosome and spectrosome are complementarily involved in the spindle orientation of the male GSCs. Our investigation stemmed from a result that the majority of the spindle was still oriented despite of the centrosome being knocked out [22]. Another clue was provided when majority of the interphase spectrosomes switched locations from basal to apical cortices in *DSas4-mut* GSCs [23], where the spectrosomes were seen previously anchoring the spindle pole to orient the spindle in the wild type female GSCs. Contrary to the female GSCs, however, when the spectrosome was compromised through an *hts-mut*, male GSCs with intact centrosomes had minimally altered the centrosome and spindle orientation [23]. We propose that in male GSCs, spectrosome is recruited as a fail-safe mechanism to the apical cortex to facilitate proper spindle orientation when centrosome’s function is compromised.

## Materials and Methods

### Fly husbandry and strains

All fly stocks were raised on Bloomington medium at 25°C. Fly stocks used were: Short Adducin GFP (obtained from Dr. Spradling’s Laboratory); Ubi-α-tubulin-GFP, Ubi-Sas6-mcherry, Df(2R)BSC26, hts^01103^, Dsas-4^S2214^, FRT82B DSas-4^S2214^[21]; Df(2R)BSC26; UAS-mCD8-GFP, hs-FLP; GAL4.nos.NGT40, hts^01103^; FRT82B, GAL80LL3. Stocks were obtained from the Bloomington stock center unless otherwise noted. Mosaic Analysis with a Repressible Cell Marker (MARCM) method [24, 25] was used to generate centrosome and spectrosome knockout flies.

### Immunofluorescence microscopy

Immunofluorescence staining protocol was used as described previously [11]. The primary antibodies used were: mouse anti-fasciclin III [1:80; obtained from the Developmental Studies Hybridoma Bank (DSHB)], mouse anti-γ-tubulin monoclonal (1:80; GTU-88; Sigma), mouse anti-Adducin-like monoclonal (1:100, obtained from DSHB), goat-anti-Vasa polyclonal (1:80; dc-13; Santa Cruz Biotechnology). Images were captured using a Zeiss Axio Observer. Z1 with Apotome and Axiovision (Zeiss).

### Time-lapse live-cell imaging

Time-lapse live-cell imaging protocol was used as described previously [26]. Time-lapsed image sequences were acquired for up to 8 hours with 1 to 2 minute time intervals or 24 hours with 8 minute time intervals. Mitotic spindle were visualized by Ubi-α-tubulin-GFP, centrosome by Sas-6-mcherry, and spectrosome by Short Adducin-GFP.

### Subcellular Organelle Tracking, image processing, and dynamics quantification

Migration patterns of centrosome and spectrosome were obtained by analyzing the time lapse image sequences. Firstly, z-stack images were overlaid and the contrast was automatically enhanced (program developed in MatLab). Then the target organnelles were tracked by the pattern matching software (custom-developed in Labview). To compensate for the slow tissue drifting during observation period, the location of stem cell niche were tracked at the same time. After positions of organelles and the hub-GSC interface were obtained, more dynamic parameters were calculated: mitotic spindle enlongation velocity, GSC-inherited/GB-inherited centrosome velocity, mitotic spindle angular velocity, GSC-inherited and GB-inherited centrosome distance to the hub-GSC interface, apical to GB-inherited centrosome distance, spectrosome velocity, and spectrosome distance to the hub-GSC interface.

### Statistical Analysis

Student’s t-test was used to calculate the p-values to determine significant differences between groups. The histograms were generated by selecting a fixed number of bins that covers the range of 0 to maximum values in groups. Bin sizes used for the centrosome velocity and centrosme distance were 0.15μm/min and 0.84μm, respectively.

## Results

### Spindle orientation is maintained in most male GSCs without centrosomes

**Fig 1:**
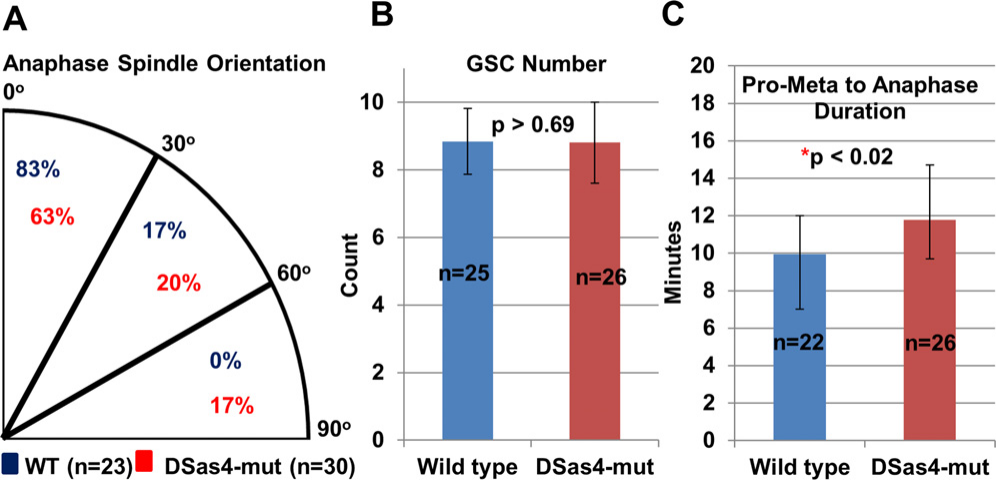
Most spindle orientation at anaphase and stem cell number are maintained in GSCs without centrosomes. **(A)** Live imaging reveals that most *DSas4-mut* GSCs maintain their spindle orientation compared to the wild type. **(B)** There is no significant difference of GSC number per testes in *DSas4-mut* and wild type flies. **(C)** The mitosis duration from pro-metaphase to anaphase in *DSas4-mut* GSCs is significantly longer than that in wild type.

Previous literature has reported that centrosomes in *Drosophila* appear to have mixed roles for some types of stem cells in maintaining asymmetric stem cell divisions. A neuroblast without centrosome due to *DSas4-mut* displays asymmetric division defects [27], but most male and female GSCs in *DSas4-mut* can still maintain asymmetric stem cell division with proper spindle alignment [21, 22]. Consistently, we found that most acentrosomal GSCs with *DSas4-mut* maintained proper orientation (Fig 1A: 63% at 0–30 degrees, 20% at 30–60 degrees, and 17% at 60–90 degrees) compared to wild type (83% at 0–30 degrees, 17% at 30–60 degrees, and 0% at 60–90 degrees). Additionally, by counting the GSC numbers per testis, we found that there is no significant difference (p>0.69) of GSC number per testis in *DSas4-mut* (8.8±1.2 GSCs, n = 26 testes) and wild type (8.8±1.0 GSCs, n = 25 testes) (Fig 1B).

To further gain an insight of spindle dynamics during mitosis, time-lapse live-cell imaging (hence forth referred as live imaging) microscopy was utilized to examine the mitotic spindle morphology and movement in GSCs. We were able to compare the effect of centrosome knockout on mitotic duration by measuring the time from the onset of nuclear envelope break down (pro-meta phase) to the beginning of spindle elongation (Anaphase). Our results showed that the time in *DSas4-mut* GSCs (11.8±2.9 minutes, n = 26) was significantly longer (p<0.02) compared to that in wild type GSCs (10.0±2.1 minutes, n = 22) (Fig 1C). These results suggest that although centrosomes are not required to maintain proper asymmetric GSC divisions, centrosomes play a role in facilitating GSC mitosis.

### Spectrosome migration pattern changes in acentrosomal GSCs

**Fig 2:**
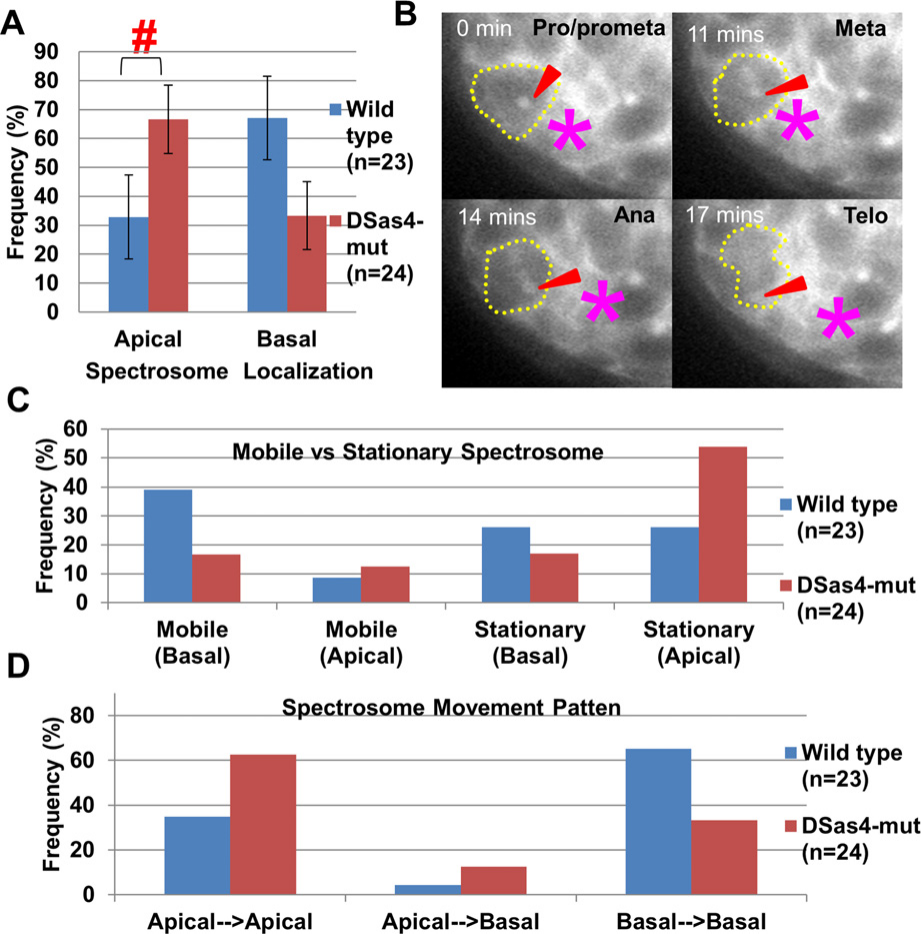
Dynamic migration patterns of spectrosomes are quantified utilizing time-lapse live-imaging. **(A)** Spectrosome localization in *DSas4-mut* becomes predominantly apical compared to wild type. #: p<0.05. **(B)** Live image sequences shows apically (to the hub cells) migrating spectrosome in a dividing *DSas4-mut* GSC. Arrowhead: spectrosome. *: hub cells. Yellow dash-line: GSC. **(C)** In wild type GSCs, large majority of spectrosomes were mobile and located basally (39%) during interphase. In *DSas4-mut* GSCs, majority of spectrosomes were stationary and located apically (54%). Wild type: n = 23, DSas4-mut: n = 24. **(D)** *DSas4-mut* GSCs had higher percentage of spectrosomes migrating from apical to the basal side prior to mitosis. Spectrosome switches are categorized as such if they migrate within 30 mins prior to mitosis (identified by nuclear envelope breakdown).

Although previous fixed sample study showed that the spectrosome in male GSCs had higher frequency localizing at the apical cortex in *DSas4-mut* than that in wild type [23], the migration pattern of spectrosome remain unknown. To better understand the migration of the spectrosome, live imaging study was used to examine the movement pattern of spectrosome. Firstly, results from the live imaging show that spectrosome frequently localized to the apical region when the centrosome was knocked out in *DSas4-mut* (67 ± 12%, n = 24) compared with wild type (33 ± 14%, n = 23) (Fig 2A). Additionally, live imaging revealed previously undiscovered dynamic movement of spectrosome (a typical *DSas4-mut* GSC is shown in Fig 2B). Spectrosomes in both wild type and *DSas4-mut* were mobile during interphase and became immobilized prior to entering mitosis. In wild type (n = 23) GSCs, mobile and basally positioned spectrosomes composed 39%, while stationary and apically positioned spectrosomes composed 26% (Fig 2C). In *DSas4-mut* (n = 24) GSCs, stationary and apically positioned spectrosomes composed majority at 54%, while mobile and basally positioned spectrosomes composed 17%. Spectrosome was counted as stationary when it stays at either basal or apical ends for 30 minutes or longer. Furthermore, spectrosomes are highly positioned at the apical region (75%, 3 out of 4 GSCs) in the very rare and severely misoriented spindles in *DSas4-mut* GSCs, while only 54% (13 out of 24 GSCs) positioned at apical region in *DSas4-mut* GSCs with the properly oriented spindles. On few occasions, the apically located spectrosomes quickly migrated over to the basal location prior to mitosis (see S1 Movie) (wild type: 4%, *DSas4-mut*: 13%) (Fig 2D). These results demonstrate that without centrosome, majority of spectrosomes position at the apical end of the GSC and become immobilized.

### Spectrosome material transfers between daughter cells during asymmetric GSC divisions

**Fig 3:**
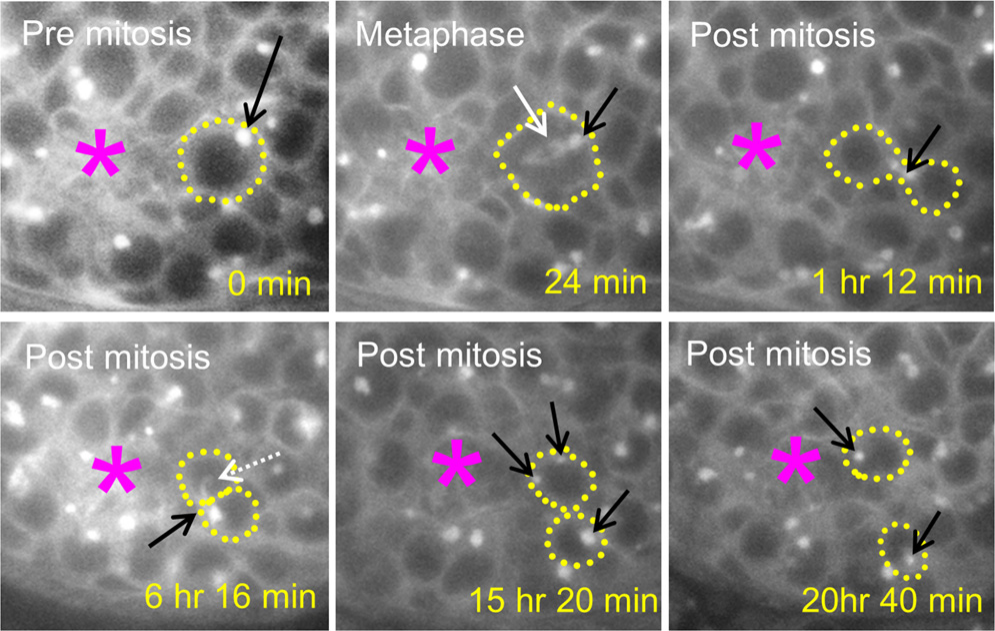
Spectrosome material is transferred via ring canal in GSCs. Spectrosome (Short Adducin-GFP) in wild type GSC is located in the basal position (0 min: interphase and 24min: metaphase). At 1hr 12min, spectrosome is co-localized with the ring canal structure. Spectrosome, together with ring canal structure, grows into the stem cell daughter at 6hr 16min, segregates into both daughter cells and form spectrosome-like structure again at 15hr 20 min. At 20hr 40 min, spectrosomes in both GSC and gonialblast further develop, and the gonialblast separates from GSC. Cellular boundaries and mitotic spindle are visualized with α-tub-GFP. Black arrow: spectrosome. White dash-arrow: spectrosome and/or ring canal tail. *: hub cells. Yellow dash-line: GSC or gonialblast boundary. Solid white arrow: mitotic spindle.

Next, we investigated, during asymmetric GSC division, how the spectrosome is dynamically transferred among the two daughter cells. Prior to completion of cytokinesis, spectrosome can initially be received by either stem cell daughter (i.e., GSC) or the differentiating daughter cell (i.e., gonialblast or GB) depending on the positioning of the spectrosomes (apically or basally) at the onset of mitosis. The dynamic spectrosome material transfer process can be illustrated in a typical example shown in Fig 3. Prior to and during mitosis, spectrosome in wild type GSC generally remained stationary at the basal end of the GSC, as shown in Fig 3 (0 min, prior to mitosis and 24 min, metaphase). At the end of mitosis (1hr 12 mins), the spectrosome moved to and localized at the bridge between two daughter cells, presumably co-localized with the intercellular bridge known as ring canal. On wild type spectrosome live-imaging sessions, the spectrosome, whether received by the GSC or the gonialblast during mitosis, in almost all cases conjoined to the ring canal and formed a complex. Many hours later, smaller tail region of the ring canal and spectrosome complex grew and further extended into the daughter GSC (Fig 3, 6hr 16min). Finally, the ring canal and spectrosome complex structure broke apart and was transferred to both the daughter GSC and the gonialblast when the gonialblast and the GSC detached from each other (Fig 3, 15hr 20 min). Typical counts of spectrosome like structures in the GSC (Fig 3, 15hr 20 min) vary from one to multiple. Based on many observations, the gonialblast appeared to inherit larger portions of the spectrosome. The GSC and its spectrosome size further grew while the gonialblast slightly separated away from the stem cell niche (Fig 3, 20hr 40 min). These results demonstrate that ring canal plays an important role in facilitating the spectrosome transfer between the two daughter cells (GSC and gonialblast) regardless of the initial spectrosome position prior to mitosis.

### *hts-mut* does not affect centrosome orientation or mitotic spindle orientation

**Fig 4:**
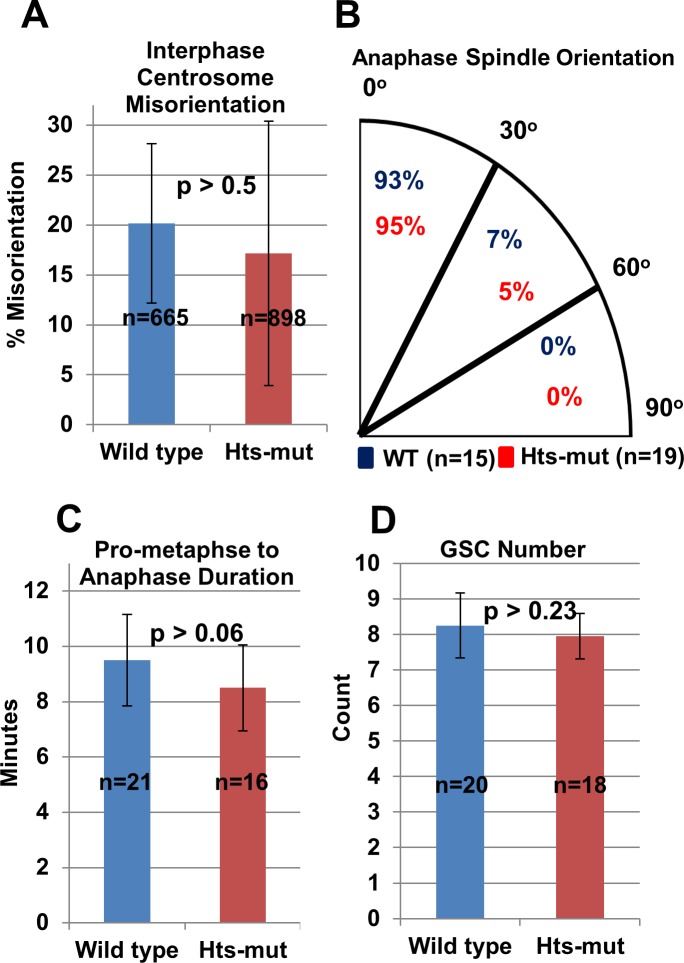
Spectrosome Knockout minimally affects centrosome orientation, spindle orientation, mitosis duration, and stem cell numbers in male GSCs. **(A)** Centrosome misorientation during interphase was analyzed from time lapse image sequences, and there is no significant difference between the *hts-mut* and the wild type. **(B)** There is minimal change in the spindle orientation between the *hts-mut* and the wild type. **(C)** Mitosis duration is not affected in *hts-mut* compared to the wild type when measured from nuclear envelope breakdown time to anaphase. **(D)** There is no significant difference for GSC number per testis in hts-mut and wild type flies.

Utilizing time-lapse live-cell imaging, we investigated the centrosome and spindle orientation when spectrosome was knocked out. Firstly, consistent with reported results based on fixed sample studies, *hts-mut* does not affect either the interphase centrosome orientation (p>0.5) (Fig 4A) or the mitotic spindle orientation, as 95% spindles in *hts-mut* were still properly oriented (Fig 4B). Moreover, the *hts-mut* does not significantly affect mitosis duration either (Fig 4C: from nuclear envelope break time to the onset of anaphase, p>0.06); wild type GSCs were 9.5±1.5min (n = 21) and *hts-mut* GSCs were 8.5±1.7min (n = 21). We found that wild type GSC count per testis (8.3±0.9 GSCs, n = 20 testes) and *hts-mut* GSC counts per testis (7.9±0.6 GSCs, n = 18 testes) were not significantly different (p>0.23), (Fig 4D). These results demonstrate that the spectrosome does not affect the centrosome and spindle orientation.

### *hts-mut* affects centrosome migration velocity and centrosome position in GSCs

**Fig 5:**
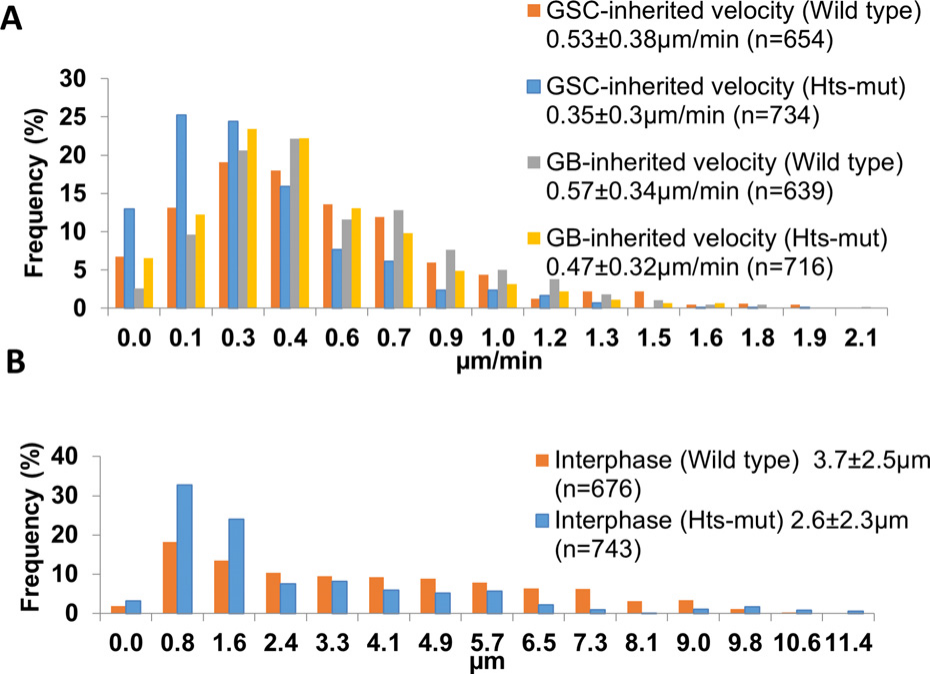
Centrosome velocity and distance to hub-GSC interface change in *hts-mut* GSCs. Based on the centrosome tracking analysis of live-image sequences, the **A)** Interphase centrosome velocities are shown for both *hts-mut* GSCs and wild type GSCs (p<0.01 between *hts-mut* and wild type for both GSC-inherited and GB-inherited centrosomes), and the **B)** GSC-inherited centrosome distance to the hub-GSC interphase histograms are shown for both *hts-mut* and wild type GSCs (p<0.01).

Although spectrosome knockout does not affect centrosome or spindle orientation, further analysis of dynamic migration pattern show the centrosome migration pattern does change. Our live imaging results showed that the motility of GSC-inherited centrosomes during interphase is significantly slower than that of GB-inherited centrosomes in *hts-mut* (p<0.01). Quantitatively, in wild type, the GSC-inherited centrosome velocity is 0.53±0.38μm/min (n = 654) while the GB-inherited velocity is 0.57±0.34μm/min (n = 639); and in *hts-mut*, the GSC-inherited centrosome velocity is 0.35±0.3μm/min (n = 734) and GB-inherited velocity is 0.47±0.32μm/min (n = 716) (Fig 5A). Furthermore, GSCs in *hts-mut* flies have both less motile GSC-inherited and GB-inherited centrosomes than that in wild type (p<0.01). Interestingly, we also found that GSC-inherited centrosomes in wild type throughout the interphase were located significantly further away from the hub-GSC interface than that in *hts-mut* (p<0.01) (Fig 5B). The results reveal the previously undiscovered dynamic interplay of centrosome and spectrosome.

### Centrosome and Spectrosome double knock out using Mosaic Analysis with a Repressible Cell Marker (MARCM) Method

To further study the potential interactive roles of centrosome and spectrosome, we tried to double knockout both centrosomes and spectrosome. Because the *DSas4-mut* and *hts-mut* double mutation is lethal, MARCM method was adapted to generate *DSas4-mut* GSC clones on *hts-mut* background. Using the MARCM method, we successfully obtained double mutant clones, however no mitotic double mutant clone was observed (out of 65 double mutant clones). Considering the typical frequency of observing mitotic GSCs in wild type is about 1 per 5 testes, this result suggest that the mitotic activity is significantly suppressed when centrosomes and spectrosomes both malfunction.

## Discussions

Centrosome is known as the microtubule organization center, facilitating mitotic spindle formation during mitosis. Our and previous results show most spindle in male GSCs were still properly oriented despite of lack of centrosome, implying, when centrosome is knocked out, another regulatory mechanism would ensure proper spindle orientation. One intriguing discovery is that, the spectrosome migrates from basal to apical in most acentrosomal GSCs, which is the stereotypical location for female GSCs where spectrosome has been shown to play an important role in orienting mitotic spindles [20]. In the wild type GSCs, the spectrosomes were seen to be about half mobile and half stationary, but when the centrosomes were knocked out, close to 71% of spectrosome became stationary, and stationary spectrosomes locating at the apical end increased dramatically. The higher frequency of stationary spectrosome locating closer to the apical region would imply they may interact with the apical mitotic spindle pole and/or the apical cortex. Spectrosome organelle itself physically anchors or provides components necessary to orient the spindle [20], and thus it migrates to the apical in male GSCs when centrosome is absent. Additionally, it is reported previously that spectrosome and fusome (elongated structure with similar components as spectrosome in 4-cell spermatogonia or later) have transport capability for various proteins [13, 19, 23, 28]. Moreover, based on live imaging observations, most *DSas4-mut* GSCs still end up dividing asymmetrically even when their spindles are not properly oriented. Consistent with this observation, the GSC number per testis in *DSas4-mut* is not significantly different compared to that in wild type. Nevertheless, absence of centrosome significantly prolonged the GSC mitosis duration.

In female GSCs, the spectrosome is mostly found in the apical position during interphase, and is unequally cleaved and segregated during telophase [13, 20, 28, 29]. However, the growth and migration of spectrosome in male GSCs had not been reported. Here, we observed the spectrosome growth and migration in live male GSCs, and found that no cleavage of the spectrosome occurred at early stages during mitosis in wild type GSCs. Instead, our live imaging data in male GSCs (both wild type and *DSas4-mut*) show that spectrosomes initially retained in either GSC daughter or GB daughter based on its initial position, most moved and conjoined with the ring canal prior to cytokinesis completion, segregated together with the ring canal structure when the two daughter cells detached from each other. It is an interesting finding that spectrosome eventually transfers into both GSC and GB regardless of initial positions (apical or basal).

We were naturally curious how the spectrosome coordinate with centrosome in terms of orienting mitotic spindle. The live imaging study with spectrosome knockout provides valuable hints to the centrosomes migration pattern (mostly in G2-M cell cycle phases). There are significant differences in the centrosome velocities of GSC-inherited centrosomes and GB-inherited centrosomes in wild type compared to the *hts-mut*. The slower GSC-inherited centrosome can be interpreted by more robust microtubule arrays around GSC-inherited centrosome [10]. Also, this may imply that the GSC-inherited centrosome in general might contain different composition materials [30], contributing to smaller centrosome mobility. These results suggest spectrosome coordinates with centrosomes to ensure centrosome orientation, but without the spectrosome, another fail safe mechanism intervenes to ensure centrosome orientation.

Consistent to the results reported by Yuan et al., 2012, we observed insignificant changes in the interphase centrosome orientation and almost no spindle orientation change in spectrosome knockout GSCs. In addition, spectrosome knockout does not affect the mitotic duration. To better understand the spectrosomes’ role in the absence of centrosomes, we tried to employ the MARCM method to create the double knockout in GSC clones. This MARCM method was a promising alternative to the actual double mutant animal model since an attempt to generate double knockout adult flies failed previously. Due to the technical challenges, we were unable to observe any mitotic GSCs albeit examining 65 double-knockout GSC clones, implying that double mutant GSCs have an extremely low mitotic activity.

In summary, we characterized the dynamic movement of centrosome and spectrosome during asymmetric GSC divisions in wild type, and showed how the dynamics changed when either centrosome or spectrosome is knocked out. Based on our results, we propose that the dynamic interplay of spectrosome and centrosome is part of the regulatory mechanisms of the GSCs, to compensate for the loss of function of the knocked out organelles, which were intended to ensure an asymmetric division of the GSCs.

## Supporting Information

S1 Movie Spectrosome switches sides from the apical to the basal side prior to mitosis in a *DSas4-mut* GSC. A spectrosome is shown to migrate from the apical side (9 min) to the basal side (21 min) of a *DSas4-mut* GSC during interphase, and moves to the gonialblast (40 min). This movie ends soon after telophase so that it does not contain later events, such as spectrosome conjoining to the ring canal and spectrosome transfer between gonialblast and the GSC. Red arrowhead: spectrosome. *: hub cells. Yellow dash-line: GSC or gonialblast boundary.
(AVI)

S2 Movie Spectrosome is mostly found in the basal side in control GSCs. A representative spectrosome is shown consistently at the basal side of a *DSas4-mut/*+ GSC. Red arrowhead: spectrosome. *: hub cells. Yellow dash-line: GSC or gonialblast boundary.
(AVI)

S3 Movie Spectrosome is mostly found in the apical side in *DSas4-mut* GSCs. A representative spectrosome is shown at the apical side of the *DSas4-mut* GSC. Red arrowhead: spectrosome. *: hub cells. Yellow dash-line: GSC or gonialblast boundary.
(AVI)

S4 Movie Centrosome migration pattern in control GSCs. A representative centrosome migration pattern in *Hts-mut*/+ GSC is shown. Orange arrowhead: apical centrosome. Blue arrowhead: basal centrosome *: hub cells. Yellow dash-line: GSC or gonialblast boundary.
(AVI)

S5 Movie Centrosome migration pattern in *Hts-mut* GSCs. A respresentative centrosome migration pattern in *Hts-mut* GSC is shown. Orange arrowhead: apical centrosome. Blue arrowhead: basal centrosome *: hub cells. Yellow dash-line: GSC or gonialblast boundary.
(AVI)

S6 Movie Centrosomes and spectrosome migration pattern in wild type GSCs. A respresentative migration pattern of centrosome (sas6-mcherry) and spectrosome (shadd-GFP) in wildtype GSC is shown. Orange arrowhead: apical centrosome. Blue arrowhead: basal centrosome. Red arrowhead: spectrosome *: hub cells. Yellow dash-line: GSC or gonialblast boundary.
(AVI)
